# Curcumin blocks autophagy and activates apoptosis of malignant mesothelioma cell lines and increases the survival of mice intraperitoneally transplanted with a malignant mesothelioma cell line

**DOI:** 10.18632/oncotarget.14907

**Published:** 2017-01-30

**Authors:** Laura Masuelli, Monica Benvenuto, Enrica Di Stefano, Rosanna Mattera, Massimo Fantini, Giuseppina De Feudis, Enrico De Smaele, Ilaria Tresoldi, Maria Gabriella Giganti, Andrea Modesti, Roberto Bei

**Affiliations:** ^1^ Department of Experimental Medicine, University of Rome “Sapienza”, Rome, Italy; ^2^ Department of Clinical Sciences and Translational Medicine, University of Rome “Tor Vergata”, Rome, Italy; ^3^ Center for Regenerative Medicine, (CIMER), University of Rome “Tor Vergata”, Rome, Italy

**Keywords:** curcumin, malignant mesothelioma, apoptosis, autophagy, proliferation

## Abstract

Malignant mesothelioma (MM) is a primary tumor arising from the serous membranes. The resistance of MM patients to conventional therapies, and the poor patients’ survival, encouraged the identification of molecular targets for MM treatment. Curcumin (CUR) is a “multifunctional drug”. We explored the *in vitro* effects of CUR on cell proliferation, cell cycle regulation, pro-survival signaling pathways, apoptosis, autophagy of human (MM-B1, H-Meso-1, MM-F1), and mouse (#40a) MM cells. In addition, we evaluated the *in vivo* anti-tumor activities of CUR in C57BL/6 mice intraperitoneally transplanted with #40a cells forming ascites.

CUR *in vitro* inhibited MM cells survival in a dose- and time-dependent manner and increased reactive oxygen species’intracellular production and induced DNA damage. CUR triggered autophagic flux, but the process was then blocked and was coincident with caspase 8 activation which activates apoptosis. CUR-mediated apoptosis was supported by the increase of Bax/Bcl-2 ratio, increase of p53 expression, activation of caspase 9, cleavage of PARP-1, increase of the percentage of cells in the sub G1 phase which was reduced (MM-F1 and #40a) or abolished (MM-B1 and H-Meso-1) after MM cells incubation with the apoptosis inhibitor Z-VAD-FMK. CUR treatment stimulated the phosphorylation of ERK1/2 and p38 MAPK, inhibited that of p54 JNK and AKT, increased c-Jun expression and phosphorylation and prevented NF-κB nuclear translocation. Intraperitoneal administration of CUR increased the median survival of C57BL/6 mice intraperitoneally transplanted with #40a cells and reduced the risk of developing tumors. Our findings may have important implications for the design of MM treatment using CUR.

## INTRODUCTION

Malignant mesothelioma (MM) is a primary tumor arising from the mesothelial cell linings of the serous membranes, most commonly involving the pleural and peritoneal spaces [1]. MM generally presents as an epithelioid phenotype, although biphasic or sarcomatoid phenotypes can occur [2–3]. Mesothelium carcinogenesis is a multi-step process arising from genetic alterations that drive the progressive transformation of normal mesothelial cells into MM [4]. The development of MM has been linked to the exposure to asbestos causing random chromosome breaks [5]. Asbestos fibers can also induce non-genotoxic damages, including the abnormal activation of the AP-1/TNF-α/NF-κB autocrine pathway, which increases cell survival after DNA damage and promotes uncontrolled cell growth [6]. After the fibers’ damage to the mesothelium integrity, macrophages are activated in an attempt to remove the fibers [6–8]. These events trigger a long-lasting inflammation, which can enhance DNA damage for the production of free radicals and inflammatory cytokines [9]. The long-lasting inflammation caused by MM and the poor response to therapeutics might be due to the ability of MM cells to subvert host immune response [10, 11]. The knowledge of MM pathophysiology might influence MM patients therapy and survival [4, 12–14]. However, despite this knowledge, the MM patients’ survival is poor, and was slowly improving in the last decades [15]. Recently, the application of hyperthermic intraperitoneal chemotherapy (HIPEC) and cytoreductive surgery, had increased MM patients’ survival in particular for peritoneal mesothelioma [12]. However, the therapeutic strategies for the treatment of malignant pleural mesothelioma are referred to as ‘life-extending treatments’ [16]. The drugs employed over the past decades have shown poor response rate (RR), often no higher than 20% [16]. The resistance of MM to conventional therapies, and the poor patient survival following traditional chemotherapy, have supported the identification of novel molecular targets for MM treatment. Preclinical studies have employed a second generation of drugs including inhibitors of mTOR, folate, receptor tyrosine kinase and ciclooxygenase [16]. Clinical trials have been performed using chemotherapy with proteasome, mTOR and histone deacetylases inhibitors [16]. However, although targeted therapies improved the patients’ quality of the life and survival and the new generation of folate inhibitors alone or in combination with platinum derivatives, produced encouraging results, the absolute RRs continued to be limited compared to other tumors [16]. Accordingly, since cancer cells show aberrant signaling pathways, it might be important to employ compounds that are able to target these multiple abnormally activated transduction pathways. Polyphenols can be employed to inhibit the growth of cancer cells due to their ability to down-regulate or block the activity of multiple targets involved in carcinogenesis [17–21]. Curcumin (CUR) CUR [l,7-bis-(4-hydroxy-3-methoxyphenyl)-l,6-heptadiene-3,5-dione], is a non-flavonoid polyphenol found in the plant *Curcuma longa*, widely employed as a food additive as well as in cosmetic and herbal medicine in Asia [22–25]. Due to its ability to modulate the activity of multiple targets involved in carcinogenesis, CUR is considered a “multifunctional drug” [22–25]. Few reports described the use of CUR to inhibit the growth of MM cells. CUR induced cell death by pyroptosis in mouse and human malignant mesothelioma cells [26]. Yamauchi *et al*. reported that CUR induced autophagy in ACC-MESO-1 cells [27]. In addition, Mayol *et al*. reported that CUR loaded PLGA-poloxamer blend nanoparticles induced cell cycle arrest in MM cells [28]. Only two reports investigated the *in vivo* effect of CUR on solid tumor burden in mouse models of MM [26, 29]. Little is known about the effect of CUR on signal transduction pathways activated in MM cells and on the *in vivo* growth of MM cells. Thus, it would be essential to further investigate the *in vivo* effect of CUR in a mouse model in which MM cells induce ascites in the peritoneal space.

In this report, we explored the *in vitro* effects of CUR on cell proliferation, cell cycle regulation, pro-survival signaling pathways, apoptosis and autophagy in human and mouse MM cell lines. In addition, we evaluated the *in vivo* antitumor activities of CUR in C57BL/6 mice intraperitoneally transplanted with mouse MM cells inducing ascites.

## RESULTS

### Curcumin inhibits human and mouse MM cells survival

**Figure 1:**
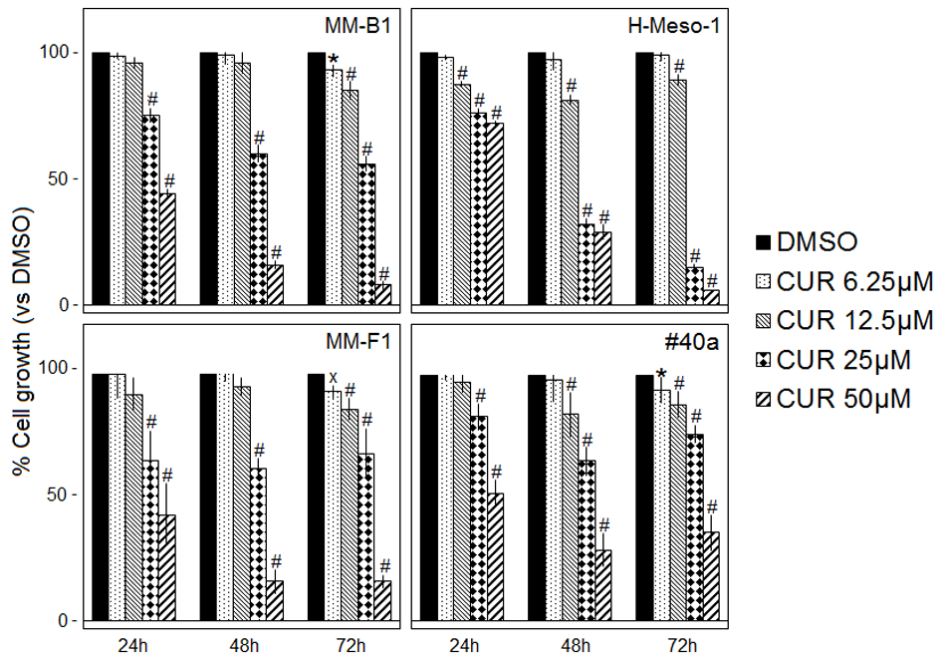
Effect of CUR on MM cell lines survival The survival of human (MM-B1, H-Meso-1, MM-F1) and mouse (#40a) cell lines was assessed by the SRB assay after 24, 48 and 72 hours of treatment with DMSO or CUR. The percentage of surviving cells treated with the compound was calculated by normalizing the O.D. value to that of the control cultures (DMSO). The results are expressed as the means ± SD of three independent experiments performed in triplicate (^x^p ≤ 0.05, *p ≤ 0.01, #p ≤ 0.001 compared with the cultures treated with DMSO).

The survival of human (MM-B1, H-Meso-1, MM-F1) and mouse (#40a) MM cells was evaluated by the SRB assay after exposure to increasing doses of CUR (6.25-12.5-25-50 μM) or vehicle control (DMSO) for 24, 48 and 72 hours (Figure 1). The effect of CUR on cell proliferation was dose- and time-dependent and was significant compared with that of the vehicle control at higher doses. CUR treatment of the MM-B1, MM-F1 and the #40a cell lines for 72 hours was able to significantly inhibit MM cell growth even at the lowest concentration (Figure 1).

The concentration of the compound that inhibits 50% of cell growth (IC50) was also determined. The concentrations of CUR required to reduce cell survival by 50% after 48 and 72 hours were 28.85 and 25.73 μM for MM-B1, respectively; 22.21 and 18.38 μM for H-Meso-1, respectively; 29.45 and 30.47 μM for MM-F1, respectively and 33.13 and 40.92 for #40a, respectively (Table 1).

**Table 1:** CUR concentration required for 50% inhibition of MM cell lines survival (IC50)

MM cell lines	CUR treatment (hours)	IC50 (μM) ± SD
**MM-B1**	48	28.85 ± 1.40
	72	25.73 ± 1.53
**H-Meso-1**	48	22.21 ± 1.39
	72	18.38 ± 0.20
**MM-F1**	48	29.45 ± 0.83
	72	30.47 ± 3.68
**#40a**	48	33.13 ± 4.18
	72	40.92 ± 4.42

### Curcumin induces reactive oxygen species (ROS) generation in MM cells

One of the major detrimental effects of CUR on cancer cells is its ability to increase ROS [30, 31]. To determine the effect of CUR on intracellular ROS production, the DCF-DA assay was performed in CUR-treated MM cells. The effects of the compound were compared to those of DMSO and the results were expressed as the mean of the fluorescence intensity (Table 2). CUR induced a significant dose-dependent ROS production as compared to the vehicle in all MM cells.

**Table 2:** Effects of CUR on the intracellular ROS production in MM cell lines

	MM-B1		H-Meso-1		MM-F1		#40a	
	**Mean±SD^a^**	**p**	**Mean±SD**	**p**	**Mean±SD**	**p**	**Mean±SD**	**p**
**DMSO**	3744±212		4223±119		5580±31		3234±307	
**CUR 6.25**	4362±185		4817±104		5680±42		3724±709	
**CUR 12.5**	4674±314		5771±708	<0.001	6485±119	<0.001	5550±197	<0.01
**CUR 25**	7762±561	<0.001	6057±586	<0.001	7147±120	<0.001	7437±1322	<0.001
**CUR 50**	9263±931	<0.001	6328±343	<0.001	7121±61	<0.001	8150±1176	<0.001

^a^The results are reported as the mean of the fluorescence intensity ± SD values from three experiments performed in triplicate. CUR was used in the range 6.25-50 μM. The statistical significance of the effect of CUR was calculated *vs*. that of DMSO.

**Figure 2:**
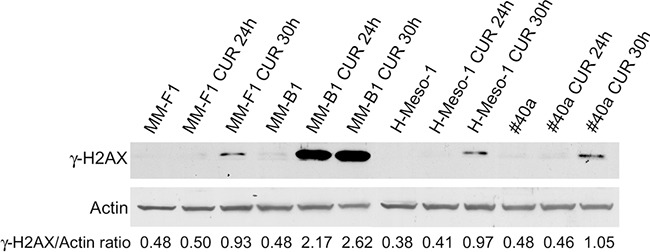
Effect of CUR on DNA damage in MM cells The expression of γ-H2AX was assessed by western blotting in MM cell lines treated with CUR at 25 μM or with DMSO for 24 and 30 hours. Actin was used as an internal control. The intensity of the bands was quantified using the ImageJ software after blot scanning of two independent experiments. The densitometric ratios between γ-H2AX and actin are reported.

ROS generation is supposed to cause DNA damage that rapidly results in the phosphorylation of the histone H2A variant (H2AX) at Ser 139 (γ-H2AX) [32, 33]. Treatment with CUR at the concentration of 25 μM for 30 hours led to a significant increased phosphorylation of γ-H2AX in all MM cell lines (MM-F1, p<0.01; MM-B1, p<0.001; H-Meso-1, p<0.001; #40a, p<0.01) (Figure 2).

### Curcumin increases p62 expression, impairs the autophagic flux and activates apoptosis in MM cells

**Figure 3:**
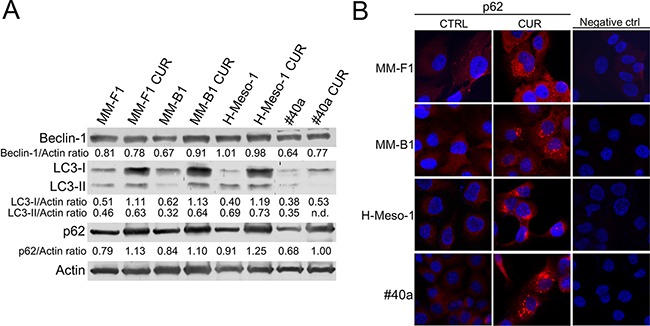
Effect of CUR on the autophagic flux in MM cells **Panel A**. The expression of Beclin-1, LC3-I and LC3-II, and p62 was assessed by western blotting in MM cell lines treated with CUR at 25 μM or DMSO for 24 hours. Actin was used as an internal control. The intensity of the bands was quantified using the ImageJ software after blot scanning of two independent experiments. The densitometric ratios between Beclin-1 and actin, LC3-I and actin, LC3-II and actin, p62 and actin are reported. **Panel B**. The expression of p62 after treatment with CUR in MM cells was determined by immunofluorescence analysis. Cells were fixed after treatment, and incubated with the anti-p62 antibody. After two washes with PBS, cells were incubated with the secondary Alexa fluor-594-conjugated goat anti-rabbit IgG antibody. Nuclei were stained with Hoechst. Original magnification x400. n.d= not detectable.

Reactive oxygen species (ROS) are the main intracellular signal transducers sustaining autophagy [34] whose activation can be revealed by the conversion of LC3-I in LC3-II. MM cells were treated with 25μM CUR or DMSO for 24 hours (Figure 3, Panel A). LC3-I and LC3-II were constitutively expressed in DMSO-treated cells. CUR induced a significant increase of LC3-I in all cells (MM-F1, p=0.0009; MM-B1, p=0.002; H-Meso-1, p=0.002; #40a, p=0.03). However the increase of LC3-I was not paralleled by a significant increase of LC3-II except for MM-B1 cells (p=0.003) that showed a significant increase of Beclin-1 as well (p=0.002). The likelihood of cancer cells toward autophagy or apoptosis is dependent on p62 expression. CUR induced a significant increase of p62 in all MM cell lines as detected by western blotting (MM-F1, p=0.004; MM-B1, p=0.02; H-Meso-1, p=0.002; #40a, p=0.004) (Figure 3, Panel A). The increase of p62 in CUR-treated MM cell lines was corroborated by immunofluorescence analysis (Figure 3, Panel B). These results indicated that CUR triggered autophagy but that the process was then blocked as revealed by the increase of p62.

**Figure 4:**
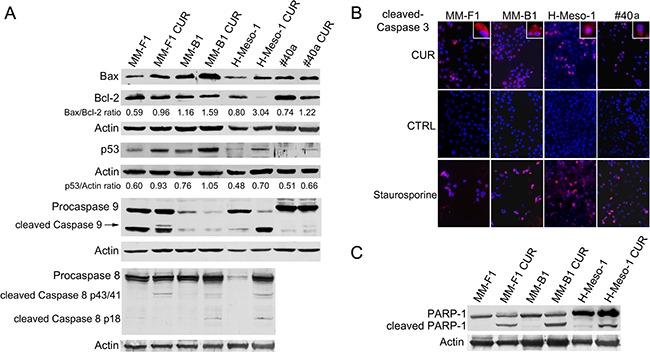
Effect of CUR on apoptosis in MM cells **Panel A**. The expression of Bax, Bcl-2, p53, procaspases (9 and 8) and caspases (9 and 8) was assessed by western blotting analysis in MM cells treated for 24 hours with CUR at 25 μM or with DMSO as vehicle. Actin was used as an internal control. The intensity of the bands was quantified using ImageJ software after blot scanning, obtained from two independent experiments. The densitometric ratios between Bax and Bcl-2, and between p53 and actin are reported. **Panel B**. The expression of active caspase 3 in MM cells treated with CUR at 25μM for 24 hours was determined by immunofluorescence analysis. Cells were fixed after treatment and incubated with the anti-cleaved caspase 3 antibody. After two washes with PBS, cells were incubated with the secondary Alexa fluor-594-conjugated goat anti-rabbit IgG antibody. Nuclei were stained with Hoechst. Staurosporine at 1 μM for 16 hours was used as positive control of apoptosis. Original magnification x200 and x400. **Panel C**. The cleavage of PARP-1 in CUR-treated MM cells. Western blotting was performed on cells treated with a concentration of 25 μM of CUR or the DMSO vehicle for 24 h. Actin was used as an internal control.

Autophagy reflects the ability of the cell to adapt to stress. However, if the stress is too powerful, the process of autophagy is bypassed and apoptosis is activated [35]. The increase of p53 represses the autophagy and activates multiple pro-apoptotic genes [35]. Thus, p53 and Bax/Bcl-2 expression was analyzed by western blotting after MM cells treatment with 25 μM CUR or DMSO for 24 hours. CUR treatment increased the Bax/Bcl-2 ratio in MM cell lines compared to DMSO treatment (MM-F1, p=0.001; MM-B1, p=0.007; H-Meso-1, p=0.002; #40a, p=0.01) (Figure 4, Panel A). In addition, CUR increased p53 expression compared to DMSO treatment in MM-F1 (p=0.012), MM-B1 (p=0.028), and H-Meso-1 (p=0.005) cells (Figure 4, Panel A). The activation of the intrinsic pathway of the apoptosis is sustained by the activation of the procaspase 9 into caspase 9. CUR was able to induce the activation of procaspase 9 in MM-B1 and #40a cells as detected by the appearance of a low molecular weight fragment of about 35 kDa, corresponding to the active caspase 9. Moreover CUR decreased procaspase 9 expression in MM-B1 and H-Meso-1 cells as compared to DMSO treated cells (ratio 0.64 vs 0.31 and 1.58 vs 0.52 respectively, p<0.05), thus suggesting the activation of the molecule [36] (Figure 4, Panel A).

In addition, we determined whether CUR was able to activate apoptosis through the extrinsic pathway as well. CUR activated procaspase 8 as detected in western blotting by the presence of caspase 8 cleavage fragments (p43/41 or p18) in human MM cells (Figure 4, Panel A).

Finally, MM cells were labeled with an anti-activated caspase 3 polyclonal antibody after treatment with CUR (25 μM) or DMSO for 24 hours or, as positive control, with staurosporine (1 μM) for 16 hours. Figure 4, Panel B shows a representative experiment. According to activated caspase 3 positivity, the treatment with DMSO had no effect on the induction of apoptosis in MM-F1 (0.006%), MM-B1 (0.006%), H-Meso-1 (0.004%) and #40a cells (0.007%) (Figure 4, Panel B). Conversely, the percentage of apoptotic CUR-treated cells was 46% for MM-F1 (p<0.001), 29% for MM-B1 (p<0.001), 53% for H-Meso-1 (p<0.001) and 34% for #40a cells (p<0.001). Treatment with staurosporine resulted in apoptotic rates of 85% for MM-F1, 79% for MM-B1, 88% for H-Meso-1 and 81% for #40a cells (Figure 4, Panel B).

**Figure 5:**
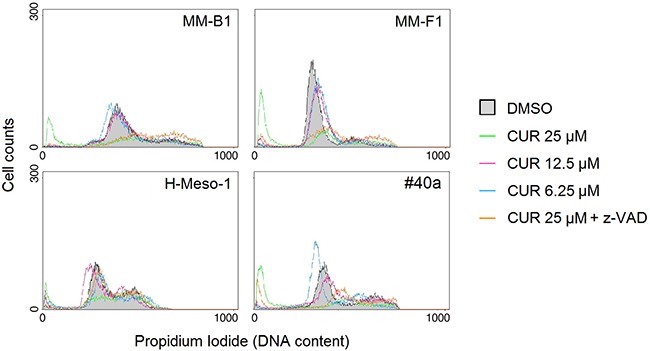
Effect of CUR on cell cycle distribution FACS analysis of DNA content was performed on asynchronized log phase growing MM cell lines treated for 48 hours with DMSO, CUR at 25, 12.5, 6.25 μM, or CUR + Z-VAD. Z-VAD-FMK (Z-VAD) is a universal inhibitor of caspases. A representative experiment is shown.

Caspase 3 cleaves poly (ADP-ribose) polymerase-1 (PARP-1) thereby inactivating it and impairing DNA repair and genomic integrity [37]. CUR-mediated cleavage of PARP-1 was determined by western blotting (Figure 4, Panel C). CUR treatment resulted in considerable PARP-1 proteolytic cleavage in all MM cells (Figure 4, Panel C). To further corroborate CUR-mediated apoptosis of MM cells, FACS analysis of DNA content was performed. Figure 5 shows a representative experiment in which the effects of increasing doses of CUR were compared to those obtained with DMSO vehicle only. Our results demonstrate that CUR induced an increase in the percentage of cells in the sub G1 phase in all MM cell lines at the higher dose (Table 3). To confirm the effect of CUR in inducing MM cells apoptosis, MM cells were simultaneously exposed to CUR and to the Z-VAD-FMK, a universal inhibitor of caspases. Z-VAD-FMK was able to significantly reduce CUR-mediated apoptosis in MM-F1 and #40a cells and abolish it in MM-B1 and H-Meso-1 cell lines (Table 3, Figure 5). The increase in the sub G1 phase mediated by CUR was associated to the increase in the G2/M and to the decrease of the G0/G1 phases in all human cell lines. The increase in the sub G1 phase was associated to a decrease of the G0/G1 phase at the dose of 25 μM in the mouse cell line (Table 3).

**Table 3:** Effects of Curcumin (CUR) alone or with the inhibitor of apoptosis Z-VAD-FMK on cell cycle distribution in MM cell lines after 48 hours of treatment

	μM	SubG1^a^	p*	G0/G1	p	S	p	G2/M	p
**MM-B1**	DMSO	6.56		68.12		7.40		18.28	
	CUR 6.25	4.96	**NS**	69.12	**NS**	8.64	**NS**	17.68	**NS**
	CUR 12.5	6.33	**NS**	64.81	**NS**	10.97	**NS**	19.39	**NS**
	CUR 25	36.05	**p<0.001**	26.42	**p<0.001**	11.20	**NS**	26.70	**p<0.05**
	CUR25+Z-VAD	6.18	**p<0.001**	24.78	**NS**	17.06	**p<0.001**	52.50	**p<0.001**
**H-Meso-1**	DMSO	2.20		59.24		9.85		29.18	
	CUR 6.25	7.39	**NS**	48.82	**NS**	9.00	**NS**	35.24	**NS**
	CUR 12.5	6.38	**NS**	60.22	**NS**	12.50	**NS**	21.51	**NS**
	CUR 25	15.09	**p<0.01**	29.19	**p<0.01**	10.39	**NS**	45.84	**p<0.05**
	CUR25+Z-VAD	4.50	**p<0.05**	51.34	**p<0.05**	11.17	**NS**	33.50	**NS**
**MM-F1**	DMSO	2.16		81.76		4.46		10.84	
	CUR 6.25	2.27	**NS**	80.26	**NS**	5.84	**NS**	11.92	**NS**
	CUR 12.5	4.36	**NS**	75.47	**NS**	6.66	**NS**	13.85	**NS**
	CUR 25	49.05	**p<0.001**	28.03	**p<0.001**	5.55	**NS**	17.58	**p<0.05**
	CUR25+Z-VAD	10.30	**p<0.001**	40.79	**p<0.01**	11.56	**p<0.05**	37.81	**p<0.001**
**#40a**	DMSO	5.63		56.98		6.96		30.60	
	CUR 6.25	6.75	**NS**	61.98	**NS**	8.17	**NS**	23.38	**NS**
	CUR 12.5	8.18	**NS**	40.92	**p<0.01**	18.78	**p<0.01**	32.61	**NS**
	CUR 25	48.24	**p<0.001**	11.07	**p<0.001**	15.76	**p<0.05**	25.30	**NS**
	CUR25+Z-VAD	18.33	**p<0.001**	13.59	**NS**	26.83	**p<0.001**	41.73	**p<0.05**

^a^ Percentage of cells in sub G1, G0/G1, S and G2/M phases was calculated with CellQuest Pro 5.2 software. The results reported are mean values from three independent experiments. * Significance of the effect of CUR was calculated vs. that of DMSO treated cells with one-way ANOVA analysis of variance. Significance of the effect of CUR25+Z-VAD was calculated vs. that of CUR 25 employing the Student's t-test.

### Curcumin increases the phosphorylation of ERK1/ERK2 and p38 but abolishes or diminishes that of JNK and AKT

**Figure 6:**
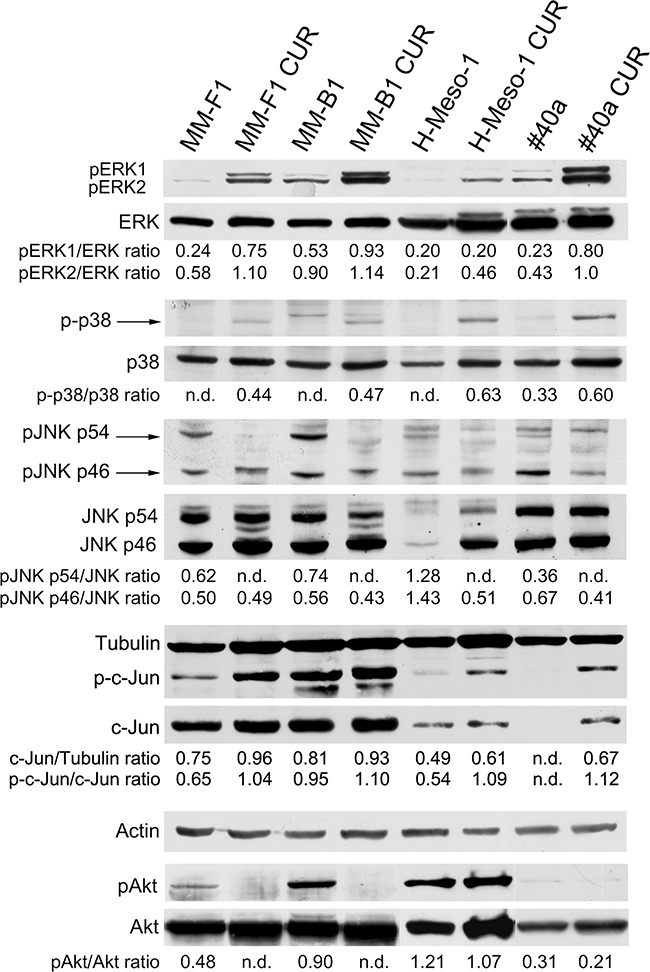
Effect of CUR on the expression and activation of signaling pathway molecules Western blotting analysis was performed on MM cells treated with CUR (25 μM) or DMSO vehicle for 24 hours. The levels of pERK1 and pERK2 proteins, as well as p-p38 protein, pJNK, p-c-Jun and pAKT proteins were compared with that of total ERK, p38, JNK, c-Jun and AKT proteins, respectively. The ratios are reported. Actin and tubulin were used as an internal control. n.d= not detectable.

It has been reported that asbestos fibers are able to activate EGFR in mesothelial cells, an event associated to the activation of the downstream extracellular signal-regulated kinases (ERKs) [38]. Thus, we evaluated the expression and phosphorylation of mitogen-activated protein (MAP) kinases including ERK1/2, the p38 kinase and the c-Jun N-terminal kinases (JNKs p54 and p46) upon CUR treatment (Figure 6). The levels of phosphorylated proteins were compared with the total proteins level. As shown in Figure 6, treatment with CUR increased the level of phosphorylation of ERK1 in MM-F1 (p=0.004), MM-B1 (p=0.017), and #40a (p=0.0004) cells compared to DMSO-treated cells. CUR treatment increased phosphorylation of ERK2 in MM-F1 (p=0.0002), MM-B1 (p=0.028), H-Meso-1 (p=0.007), and #40a (p=0.001) cells. p38 phosphorylation was increased upon CUR treatment in all MM cells (Figure 6). On the other hand, p54 JNK phosphorylation was abolished in all MM cell lines, while p46 JNK phosphorylation was decreased in MM-B1 (p=0.014), H-Meso-1(p=0.003) and #40a (p=0.0007) cells (Figure 6). Furthermore, CUR significantly increased the expression of c-Jun in all MM cell lines as compared to DMSO treated cells (MM-F1, p=0.001; MM-B1, p=0.05; H-Meso-1, p=0.02; #40a, p<0.0001). The increased expression of c-Jun was paralleled by the increase of its phosphorylation (MM-F1, p=0.001; MM-B1, p=0.01; H-Meso-1, p=0.001; #40a, p=0.0001) (Figure 6).

In addition, we evaluated whether CUR treatment inhibited the expression and phosphorylation of the pro-survival kinase AKT, which promotes tumor growth. CUR treatment abolished AKT phosphorylation in MM-F1 and MM-B1 and slightly reduced it in H-Meso-1 (p=0.047), and #40a (p=0.024) cells (Figure 6).

### Curcumin inhibits NF-κB nuclear translocation in MM cells

**Figure 7:**
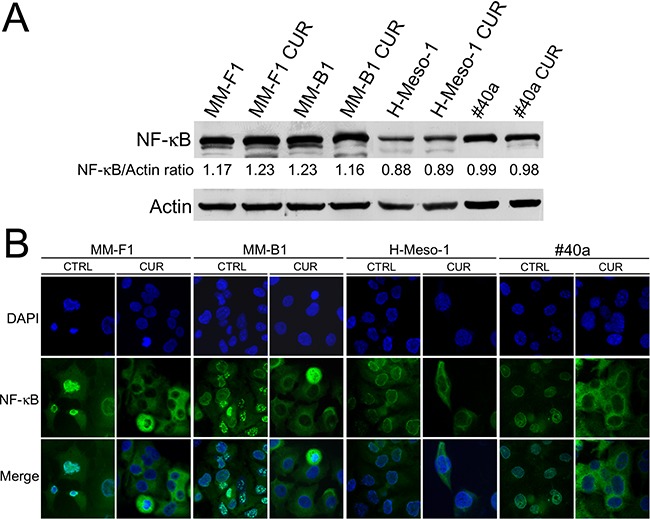
Effect of CUR on NF-κB expression and localization **Panel A**. Western blotting analysis was performed on MM cells treated with CUR at 25 μM or with the DMSO vehicle for 24 hours. The densitometric ratio between NF-κB and actin is reported. **Panel B**. Inhibition of nuclear translocation of NF-κB after treatment with CUR in MM cells was assessed by immunofluorescence analysis. Cells were fixed after treatment, and incubated with the anti-NF-κB antibody. After two washes with PBS, the cells were incubated with the secondary Alexa fluor-488-conjugated goat anti-mouse IgG antibody. Nuclei were stained with DAPI. Original magnification x400.

It was reported that JNK is able to mediate activation of NF-κB [39]. Thus, we determined whether CUR was able to modulate NF-κB activation in MM cells. Treatment with CUR did not affect the expression of NF-κB in MM cells as revealed by western blotting analysis (Figure 7, Panel A). NF-κB functions as a transcription factor, moving into its active form in the nucleus. To determine if CUR treatment was able to affect NF-κB nuclear translocation, the NF-κB localization was analyzed by immunofluorescence analysis (Figure 7, Panel B). NF-κB was found to be mainly localized in the nucleus in DMSO-MM-treated cells. Conversely, CUR treatment induced the accumulation of NF-κB in the cytoplasm of all MM cell lines, thus indicating an inhibitory effect of CUR on NF-κB nuclear translocation (Figure 7, Panel B).

### Curcumin reduces tumor growth in C57BL/6 mice intraperitoneally transplanted with MM #40a cells

**Figure 8:**
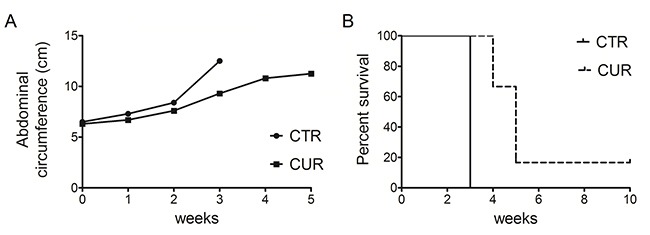
CUR reduced tumor growth and increased the survival in C57BL/6 mice intraperitoneally transplanted with MM #40a cells **Panel A**. Differences in mean tumor volumes between C57BL/6 mice treated with CUR or with corn oil (CTRL). **Panel B**. Differences in the mean survival time of C57BL/6 mice treated with CUR or with corn oil (CTRL). The numbers of inoculated mice are reported in the “Materials and Methods”.

To evaluate the *in vivo* antitumor effects of CUR, C57BL/6 mice (6 mice per group) were intraperitoneally inoculated with 1×10^6^ #40a cells. These mice were simultaneously intraperitoneally administered with 1.5 mg of CUR dissolved in corn oil or with the vehicle alone. The treatment was performed once a week. To monitor the growth of #40a cells which induced ascites, the measurement of the abdominal circumference of the mice was assessed prior to cells inoculation and then every week. After 3 weeks of treatment, mice treated with CUR showed a significant decrease in the abdominal circumference compared to control mice (mean value 9.3 cm compared with 12.5 cm, p=0.0008) (Figure 8, Panel A). At this stage, control mice were euthanized because of the excessive size of their tumors. Conversely, 2 and 3 CUR-treated mice were euthanized after 4 and 5 weeks of treatment, respectively. Only one CUR-treated mouse was still alive after ten weeks (abdominal circumference equal to 8.0 cm) when the experiment was completed. The increase in the median survival of CUR-treated mice was significant compared to vehicle-treated mice (p=0.0009) (Figure 8, Panel B). The risk of developing tumors in the corn oil-treated mice was 39.12 relative to the CUR-treated mice (Table 4).

**Table 4:** Analysis of the survival of C57BL/6 mice after treatment with CUR by the log-rank test (Mantel-Cox)

Variable	Contrast	Hazard Ratio	95%Hazard Ratio Confidence Limits		p Value	Median Survival (Weeks)
			**Lower**	**Upper**		
Treatment	CUR vs Corn Oil	39.12	4.481	341.6	0.0009	5 vs 3

Mice were treated with CUR or corn oil.

Overall, our results indicated specific interference with intraperitoneally transplanted MM #40a cells growth by CUR.

## DISCUSSION

The poor bioavailability of anti-cancer agents at the tumor sites affects the efficacy of chemotherapeutic treatments. In addition, the majority of the anti-cancer drugs does not achieve an effective concentration in the tumor [40]. Intratumoral drug delivery might facilitate the presence of a high concentration of the drug within the tumor and avoid the onset of side effects. This scenario becomes particularly interesting when the treatment can be applied to outside accessible tumors, including breast, head and neck, bladder cancers and mesothelioma.

Thus, the local administration of drugs in the serous cavity might be an improved strategy to treat MM [1]. Among the others, the polyphenol curcumin was shown to inhibit cancer cell growth by targeting multiple signaling pathways [24, 25, 29]. Clinical and preclinical studies have been demonstrated that CUR administration is safe [41]. The pharmacological potential of CUR is severely restricted due to its short half-life, poor bioavailability, low water solubility and absorption [42, 43]. Accordingly, we evaluated the *in vitro* and *in vivo* effects of CUR administration in MM cells. Our data demonstrated that treatment of MM cell lines with CUR was able to *in vitro* inhibit cells survival in a dose- and time-dependent manner. The inhibition of MM cells survival was paralleled by the increase of ROS intracellular production. CUR can function as an anti-oxidant or a pro-oxidant drug [31]. Low levels of ROS might induce cell proliferation and survival, while increased levels of ROS might induce autophagy and apoptosis by damaging DNA, proteins, and lipids [44]. DNA damage rapidly results in the phosphorylation of histone H2A variant (H2AX) at Ser 139 (γ-H2AX) [32, 33]. Our results demonstrated that treatment with CUR led to a significant increased phosphorylation of γ-H2AX in all the MM cell lines thus indicating CUR-mediated DNA damage. Oxidative stress is also a potent inducer of autophagy and apoptosis [44]. Thus, we next analyzed the ability of CUR treatment to induce autophagy in MM cell lines. Previous reports have demonstrated that CUR induces autophagy in several cancer cell lines [45]. CUR treatment was able to induce an increase in LC3-II/LC3-I ratio and autophagosome formation in the human malignant pleural mesothelioma ACC-MESO-1 cell line as well [27]. However, our results demonstrated that CUR treatment was able to trigger autophagy but that the autophagic flux was blocked as revealed by the increase of p62/SQSMT1. p62/SQSMT1 is a scaffold protein regulating signaling pathways involved in cell growth and proliferation. The levels of p62 usually inversely correlate with autophagic degradation [46]. Autophagy could be a pro-survival response for different tumors after anti-cancer treatments [47]. However, if the intensity or extent of cellular stress exceed the ability of the cells to repair the damages, the autophagy is inhibited and apoptosis is activated. Thus, autophagy often precedes apoptosis after cancer cells treatment with chemotherapeutic agents [35, 48]. p62/SQSMT1 is involved in the regulation of apoptosis by activating caspase 8 [49]. When autophagy is inhibited, caspase 8 dependent cell death is paralleled by the increase of p62 in cancer cells [50]. Here, we demonstrated that the inhibition of the autophagic flux triggered by CUR treatment and associated with the increased expression and cytoplasmic accumulation of p62, was also coincident with the activation of caspase 8 which activates the extrinsic apoptotic pathway. The activation of the extrinsic apoptotic pathway mediated by CUR treatment through Fas receptor activation, was previously established in other cancer cell lines [51–54]. Apoptosis can be also activated by oxidative stress-induced DNA damage, which activates p53 and different signal transducers such as p38, ERK and JNK [55]. p53 is a key regulators of the cell fate. DNA damage dramatically increases p53 expression and activation and cell cycle arrest and, if the DNA cannot be repaired, it activates the intrinsic apoptotic pathway [56]. Our results demonstrated that CUR treatment increased the Bax/Bcl-2 ratio in all MM cell lines by downregulating the expression of Bcl-2 and by upregulating the expression of Bax. In addition, CUR induced the increase of p53 expression and the activation of caspase 9, thus suggesting activation of the intrinsic pathway of apoptosis. Although p53 mutations are rare in MM, the loss of p14 in MM cells might result in the activation of the mdm2 protein and p53 destabilization [57]. Thus, it is of note that CUR upregulated p53 expression in MM cells. We also demonstrated that the activated caspase 3 cleaved PARP-1 thus corroborating that the inhibition of cell survival exerted by CUR treatment is mainly mediated by apoptosis. This result was corroborated by the incubation of MM cell lines with the specific inhibitor of apoptosis Z-VAD-FMK. Indeed, Z-VAD-FMK was able to significantly reduce and in MM-B1 and H-Meso-1 cell lines abolish the CUR-mediated MM cells apoptosis.

Some natural compounds, including resveratrol, quercetin and apigenin, require MAPKs activation, in particular ERK1/2 activation, for the induction of apoptosis [58]. In addition, oxidative stress is responsible for activation of MAPKs and p38 is important for ROS-mediated apoptosis [59]. Our results demonstrated that CUR treatment stimulated the phosphorylation of both ERK1/2 and p38 MAPK in all the examined MM cell lines. Of note, CUR inhibited p54 JNK phosphorylation while that of p46 JNK was only partly affected in H-Meso-1 and #40a cell lines. In addition, CUR inhibited AKT phosphorylation and NF-κB nuclear translocation. CUR inhibition of NF-κB and AKT signaling could prevent pro-survival signals thus inducing apoptosis [60]. It was reported that treatment of malignant pleural MM cell lines with butein interfered with the stability of the STAT3-NF-κB and decreased chemoresistance *in vitro* and *in vivo* [61, 62]. AKT activation has been demonstrated in malignant pleural mesothelioma specimens and PI3K/mTOR inhibitors significantly suppressed malignant pleural mesothelioma cell growth [63].

CUR-mediated apoptosis was dependent on JNK and p38 activation in colon cancer cell lines [64]. JNK and p38 may function in a cell type-specific manner to modulate intracellular signals that regulate proliferation, differentiation, survival and death. JNK and p38 can have antagonist effects. p38 activation is normally associated with anti-proliferative functions and in some cases can negatively regulate JNK activity in different cell types [65]. Recent studies have shown that p38 and JNK have a key role in the crosstalk between autophagy and apoptosis induced by DNA damage [66]. Moreover, it has been reported that JNK activation stimulates the autophagy by interfering with Bcl-2/Beclin interaction and that the pharmacological inhibition of JNK causes accumulation of p62 and reduces the LC3II/LC3I ratio flux and activates caspase 3. In particular, p54 JNK phosphorylation may support cell survival during cellular stress, while viral infections such as Kaposi sarcoma-associated herpes virus infections are able to inhibit p54 JNK phosphorylation, thus inhibiting autophagy and reducing cell survival [67–69]. In agreement with these results, the inhibition of p54 JNK phosphorylation by CUR further supports the block of the autophagic flux and the induction of apoptosis in MM cells. CUR-mediated activation and then impairment of autophagy and activation of apoptosis observed in MM cell lines could be due to the activation of ERK1/2 and p38 signaling, the last items responsible for the JNK inhibition.

c-Jun is an early response transcription factor that can be induced by DNA damage. Indeed, ERK activation is able to increase the expression of c-Jun by enhancing its stability. Phosphorylation of c-Jun is mainly mediated by JNK, but alternative pathways for c-Jun activation have been described [70, 71]. CUR increased the expression and phosphorylation of c-Jun in MM cells despite the inhibition of JNK, thus suggesting the activation of an alternative pathway for c-Jun phosphorylation. The increased expression of c-Jun upon CUR treatment might be due to activation of ERK [70].

Few studies have analysed the *in vivo* effect of CUR on MM cells growth. Miller *et al*. reported that daily oral administration of CUR via gavage (500 mg/kg and 2 g/kg) or three intraperitoneal injections of CUR (100-200 mg/kg) were not able to reduce the growth of #40 cells growing as a solid tumor in the peritoneal cavity [26]. Wang *et al*. analyzed the effect of daily administration of 500 mg/kg CUR by oral gavage in BALB/*c* mice subcutaneously inoculated with AB12 MM cells. They showed that CUR administration suppressed solid tumor growth [29]. Here, we demonstrated that the intraperitoneal administration of CUR reduced peritoneal #40a cells growth in C57BL/6 mice. The #40a cell line, when transplanted in the peritoneal cavity, is able to reproducibly induce ascites in C57BL/6 mice. Thus, the effect of CUR administration was revealed by measuring the abdominal circumference of the mice. Overall, the risk of developing tumors in the corn oil treated mice was 39.12 in comparison to those treated with 75 mg/kg CUR one time per week. In addition, the increase in the median survival of mice administered with CUR was superior to that of mice receiving corn oil. MM is an aggressive tumor. The ability of CUR to *in vivo* interfere with MM cell growth might offer an additional tool for MM treatment. The administration of CUR in the peritoneal space allows the increase of CUR concentration to which cancer cells are exposed. In addition, CUR is able to modulate the immune response [72]. Thus, CUR might potentiate mice immune response against MM cells.

**Figure 9:**
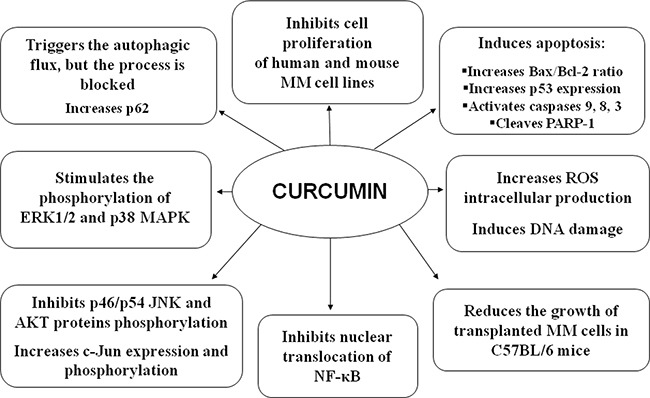
Overall *in vitro* and *in vivo* effects of CUR on MM cells

Overall, the *in vitro* and *in vivo* effects of CUR on MM cells are summarized in Figure 9.

Several *in vitro* and *in vivo* preclinical studies have brought CUR to clinical trials and its safety and efficacy has been tested and proved for using it as a chemopreventive agent or to be used in combination with the conventional chemotherapy [73]. The possibility of administering CUR directly to the tumor site could avoid the poor CUR bioavailability and potential side effects. MM patients survival is poor, although standard therapy and the incidence of MM is expected to increase in the near future. The possibility of using CUR, analogs of CUR or formulations of CUR with slower release of the compound at the tumor site could improve the delivery of the compound. Our findings may have important implications for the design of MM treatment using CUR in addition to other drugs.

## MATERIALS AND METHODS

### Reagents

DMSO, Curcumin (CUR), Sulforhodamine B (SRB), Hoechst 33342, DAPI and Pristane (2,6,10,14-Tetramethylpentadecane) were purchased from Sigma Aldrich (Milano, Italy). Z-VAD-FMK was purchased from Calbiochem (San Diego, CA, USA). Antibodies against AKT, phospho-AKT, p38 and phospho-p38, JNK and phospho-JNK, caspase 9, caspase 8, activated caspase 3, c-Jun and phospho-c-Jun were obtained from Cell Signaling Technology (MA, USA). Antibodies against Bax, Bcl-2 and γ-H2AX were obtained from BD Pharmigen (BD Biosciences, CA, USA). Antibodies against p53, PARP-1, ERK1/2 (C-14), phospho-ERK (E-4), NF-κB (p65) were obtained from Santa Cruz Biotechnology (CA, USA). Antibodies against Beclin-1 and p62/SQSTM1 were obtained from Abcam (Cambridge, UK) and the anti-LC3 antibody was purchased from Novus Biologicals (Littleton, CO, USA). Goat anti-rabbit IgG Alexa fluor-594-conjugated and goat anti-mouse IgG Alexa fluor-488-conjugated secondary antibodies were purchased from Invitrogen (Milano, Italy). The rabbit polyclonal antibody against actin and tubulin and goat anti-mouse or the anti-rabbit IgG peroxidase-conjugated secondary antibodies were obtained from Sigma-Aldrich.

### Cell lines and treatments

Human (MM-B1, H-Meso-1, MM-F1) and mouse (40 and #40a) MM cell lines were maintained in DMEM (*Dulbecco's modified Eagle's medium*) containing 10% fetal bovine serum, 100 U/ml penicillin and 100 μg/ml streptomycin (complete medium). The cells were grown at 37°C in a humidified incubator with an atmosphere of 5% CO_2_. The #40a cell line derives from the 40 cell line after two passages in the peritoneal cavity of C57BL/6 mice. These passages allow the selection of cells which reproducibly form ascites when intraperitoneally injected in the mice. H-Meso-1 cells have an epithelial morphology, while MM-B1 and MM-F1 cells have biphasic and sarcomatous features, respectively [74]. The 40 cell line has an epithelial morphology [75].

CUR was dissolved in DMSO. For the treatments, the cells were incubated for the indicated times in the presence of CUR (dose range: 6.25-50 μM) or the vehicle (DMSO ≤ 0.1%).

### Sulforhodamine B (SRB) assay

Cells were seeded at 5×10^3^/well in 96-well plates and incubated at 37°C to allow cell attachment. After 24 hours, the medium was changed and the cells were treated with CUR or DMSO and incubated for 24 hours, 48 hours, 72 hours at concentrations of 6.25-12.5-25-50 μM. The cells were then fixed with cold trichloroacetic acid (final concentration 10%) for 1 hour at 4°C. After 4 washes with distilled water, the plates were air-dried and stained for 30 min with 0.4% (wt/vol) SRB in 1% acetic acid. After 4 washes with 1% acetic acid to remove the unbound dye, the plates were air-dried, and cell-bound SRB was dissolved with 200 μl/well of 10 mM unbuffered Tris base solution. The optical density (O.D.) of the samples was determined at 540 nm with a spectrophotometric plate reader. The percentage survival of the cultures treated with CUR was calculated by normalizing their O.D. values to those of control cultures treated with DMSO [20]. The experiments were performed in triplicate and repeated three times.

### Fluorescent measurement of ROS

Dichlorofluorescin diacetate (DCF-DA) was used to detect ROS production in MM cells. Briefly, 2.5×10^5^ cells were seeded into 6-well plates and incubated at 37 °C to allow cells attachment before treatment. After two washings with PBS, cells were incubated with 10 μM 2′,7′-dichlorofluorescein diacetate (Sigma-Aldrich, Milan, Italy) in PBS at 37°C and 5% CO_2_ in the dark for 30 min [76]. After two washings, cells were treated with CUR (6.25-50 μM) or DMSO in serum-free medium and incubated at 37°C and 5% CO_2_ in the dark for different times (15 min-4 hours). Then, adherent and suspended cells were harvested, centrifuged at 1250 rpm for 10 min, and seeded in 96-well plates (100 μl per well). Fluorescence intensity was measured after 15 and 30 min and after 1 and 4 hours using a spectrophotometric plate reader at an excitation wavelength of 495 nm and an emission wavelength of 535 nm. Because the highest level of fluorescence was detected at 30 min and it decreased back to the level of the control after 1 hour of CUR stimulation (data not shown), this experimental time was chosen for subsequent experiments.

### FACS analysis

Asynchronized, log-phase growing cells (60% confluent, approximately 2.5×10^5^/well in 6-well plates) were treated with CUR (6.25-12.5-25 μM) or DMSO in complete culture medium. Z-VAD-FMK was used at a final concentration of 40 μM for 2 hours before addition of CUR treatment. After 48 hours adherent as well as suspended cells were harvested, centrifuged at 1500 rpm for 10 min and washed twice with cold phosphate-buffered saline (PBS). The cell pellets were re-suspended in 70% ethanol and incubated for 1 hour at -20°C. The cells were then washed twice with cold PBS, centrifuged at 1500 rpm for 10 min, incubated for 1 hour in the dark with propidium iodide (25 μg/ml final concentration in 0.1% citrate and 0.1% Triton X-100) and analyzed by flow cytometry using a FACSCalibur cytometer with CellQuest software [77].

### Preparation of cell lysates and western blotting

Approximately 1×10^6^ cells were seeded in 100-mm tissue culture dishes 24 hours prior to the addition of 25 μM CUR or vehicle. After 24 hours of incubation, the cells were harvested, washed twice with cold PBS and lysed in RIPA lysis buffer (Triton X-100 1%, SDS 0.1%, NaCl 200 mM, Tris HCl 50 mM pH 7.5, PMSF 1 mM, and NaOV 1 mM). After 30 min at 4°C, the mixtures were centrifuged at 12000 g for 15 min and the supernatants were analyzed by western blotting. For western blotting analysis, 50 μg of cell lysates were resolved in 10% SDS-PAGE and then transferred to nitrocellulose membranes. After blocking, the membranes were incubated with specific primary antibodies at 1-2 μg/ml concentrations overnight at 4°C. After being washed, the filters were incubated with goat anti-mouse or anti-rabbit IgG, peroxidase-conjugated antibodies and developed by chemiluminescence as previously described [78]. A densitometric analysis of autoradiographic bands was performed with Image J software (National Institutes of Health, USA) after blot scanning.

### Immunofluorescence

Cells were seeded at 4×10^4^ cells/well in 8-well chamber slides and, after 24 hours, they were treated with 25 μM CUR, or with the vehicle. After 24 hours, the cells were fixed in 4% formaldehyde for 10 min, washed and fixed in methanol for 5 min at -20°C, then washed again and incubated with specific primary antibodies for 1 hour at room temperature. After additional washings, the cells were labeled with a goat anti-rabbit IgG Alexa fluor-594-conjugated and goat anti-mouse IgG Alexa fluor-488-conjugated secondary antibody for 30 min [79]. After a third washing, the cells were incubated with 0.1 μg/ml Hoechst 33342 and mounted under a cover slip with glycerol. The cells were observed with an Olympus BX51 microscope.

### *In vivo* treatment of C57BL/6 mice intraperitoneally administered with CUR and transplanted with #40a cells

Groups of 6-to-8-weeks-old C57BL/6 mice (6 mice per group) were intraperitoneally (i.p.) inoculated with 0.2 ml of suspension containing 1.5×10^6^ #40a cells in phosphate-buffered saline (PBS) one week after pristane injection (500 μl). Mice were treated i.p. with CUR (1.5 mg dissolved in 800 μl of corn oil, 1 time per week), or corn oil (800 μl, 1 time per week). The treatments were started simultaneously with the inoculation of cells. Isolation of the murine mesothelioma 40 cell line was previously described by Goodglick *et al*. [75]. The #40a cell line is derived from the 40 cell line after two passages in the peritoneal cavity following administration of pristane one week before cell transplant. The intraperitoneal injection of a mineral oil such as pristane was shown to induce inflammation in mice [80].

Investigation has been conducted in accordance with the ethical standards and according to the Declaration of Helsinki. A veterinary surgeon was present during the experiments. The animal care both before and after the experiments was performed only by trained personnel. Mice were bred under pathogen-free conditions in the animal facilities of the University of Rome “Tor Vergata” and handled in compliance with European Union and institutional standards for animal research. The work was conducted with the formal approval of the local animal care committees (institutional and national), and animal experiments have been registered as legislation requires (Authorization from Ministry of Health n° 187/2016-PR).

### Analysis of antitumor activity *in vivo*

#40a cells growth in the peritoneum induces ascites. Accordingly, the abdominal circumference of mice was monitored before the inoculation of cells and every week until tumor-bearing mice were euthanized at the first signs of distress or when their abdominal circumference exceeded 12 cm.

### Statistical analysis

The data distribution of cell survival and the FACS analyses were preliminarily verified by Kolmogorov-Smirnov test, and data sets were analyzed by one-way analysis of variance (ANOVA) followed by Newman-Keuls test. Differences in the intensity of immunoreactive bands were evaluated by a two-tailed Student's t-test. Values with p≤ 0.05 were considered significant. Survival curves and tumor volumes were estimated using the Kaplan-Meier method and compared with a log-rank test (Mantel-Cox). Differences in tumor volumes were regarded as significant when the p value was ≤0.05 [81].

## Abbreviations

DMSO: Dimethyl sulfoxide
PARP-1: Poly (ADP-ribose) polymerase-1
ERK: extracellular signal-related kinase
p-ERK: phospho-ERK
p-AKT: phospho-AKT
MAPK: mitogen-activated protein kinase
